# Self‐Healing Hydrogels: Mechanisms and Biomedical Applications

**DOI:** 10.1002/mco2.70181

**Published:** 2025-04-24

**Authors:** Lingling Xue, Ran An, Junqi Zhao, Mengdi Qiu, Zhongxia Wang, Haozhen Ren, Decai Yu, Xinhua Zhu

**Affiliations:** ^1^ Department of Hepatobiliary Surgery Hepatobiliary Institute Nanjing Drum Tower Hospital Medical School Nanjing University Nanjing China

**Keywords:** biomedical applications, hydrogels, self‐healing mechanisms

## Abstract

Hydrogels have emerged as dependable candidates for tissue repair because of their exceptional biocompatibility and tunable mechanical properties. However, conventional hydrogels are vulnerable to damage owing to mechanical stress and environmental factors that compromise their structural integrity and reduce their lifespan. In contrast, self‐healing hydrogels with their inherent ability to restore structure and function autonomously offer prolonged efficacy and enhanced appeal. These hydrogels can be engineered into innovative forms including stimulus‐responsive, self‐degradable, injectable, and drug‐loaded variants, thereby enhancing their applicability in wound healing, drug delivery, and tissue engineering. This review summarizes the categories and mechanisms of self‐healing hydrogels, along with their biomedical applications, including tissue repair, drug delivery, and biosensing. Tissue repair includes wound healing, bone‐related repair, nerve repair, and cardiac repair. Additionally, we explored the challenges that self‐healing hydrogels continue to face in tissue repair and presented a forward‐looking perspective on their development. Consequently, it is anticipated that self‐healing hydrogels will be progressively designed and developed for applications that extend beyond tissue repair to a broader range of biomedical applications.

## Graphical abstract

This review explores the advancements in self‐healing hydrogels for tissue repair, highlighting their exceptional biocompatibility and tunable mechanical properties. It delves into the categories, self‐healing mechanisms, and biomedical applications of these hydrogels, including wound healing, drug delivery, and tissue engineering. The paper also discusses the challenges faced by self‐healing hydrogels and provides insights into their future development in the biomedical field.

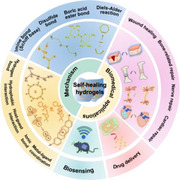

## 1 Introduction

As the population ages and the incidence of traumatic events increases, there is an increasing demand for tissue repair materials that can replace damaged tissues, facilitate natural healing, and enhance therapeutic outcomes [1, 2]. These materials, which may be either synthetic or natural, are designed to emulate the properties of the extracellular matrix (ECM) [3]. They serve as temporary scaffolds that promote cell adhesion, proliferation, and differentiation, thereby supporting the development of new tissue [4, 5]. Their applications are diverse, encompassing bone and cartilage regeneration, soft tissue and skin repair, and the reconstruction of neurons and blood vessels [6, 7, 8, 9, 10]. Hydrogels have been used as biomaterials for many years because of their distinctive properties that closely mimic the ECM of biological tissues [11, 12]. Their high‐moisture contents, biocompatibility, and customizable mechanical properties have placed hydrogels at the forefront of biomedical applications [13, 14]. However, the use of traditional hydrogels poses major challenges because of their susceptibility to mechanical failure [15]. Self‐healing hydrogels have emerged as major advancements in this domain because they address the fundamental vulnerabilities of conventional hydrogels.

Analogous to the intrinsic repair mechanisms of biological tissues, self‐healing hydrogels represent an innovative category of materials capable of autonomously restoring structural integrity and functionality after damage [16, 17]. The incorporation of dynamic chemical bonds or physical interactions into their design is essential for endowing hydrogels with self‐healing capabilities [18]. This not only reinstates the original form and functionality of the material but also extends its operational lifespan, thereby diminishing the necessity for frequent replacement. Because of their inherent biocompatibility, biodegradability, and self‐healing attributes, self‐healing hydrogels surpass traditional wound dressings and tissue‐engineered scaffolds in performance [19, 20]. Their unique three‐dimensional network structure enables the formulation of hydrogels that can encapsulate and release therapeutic agents in response to external stimuli, such as light [21], heat [22], or pH [23], thus facilitating tissue repair [24]. Moreover, these hydrogels can be engineered to exhibit shear‐thinning behavior, robust mechanical properties, and injectability, thereby catering to a diverse array of biomedical applications [25, 26]. These advantages make self‐healing hydrogels promising candidates for advances in wound care, drug delivery, and tissue engineering.

**FIGURE 1:**
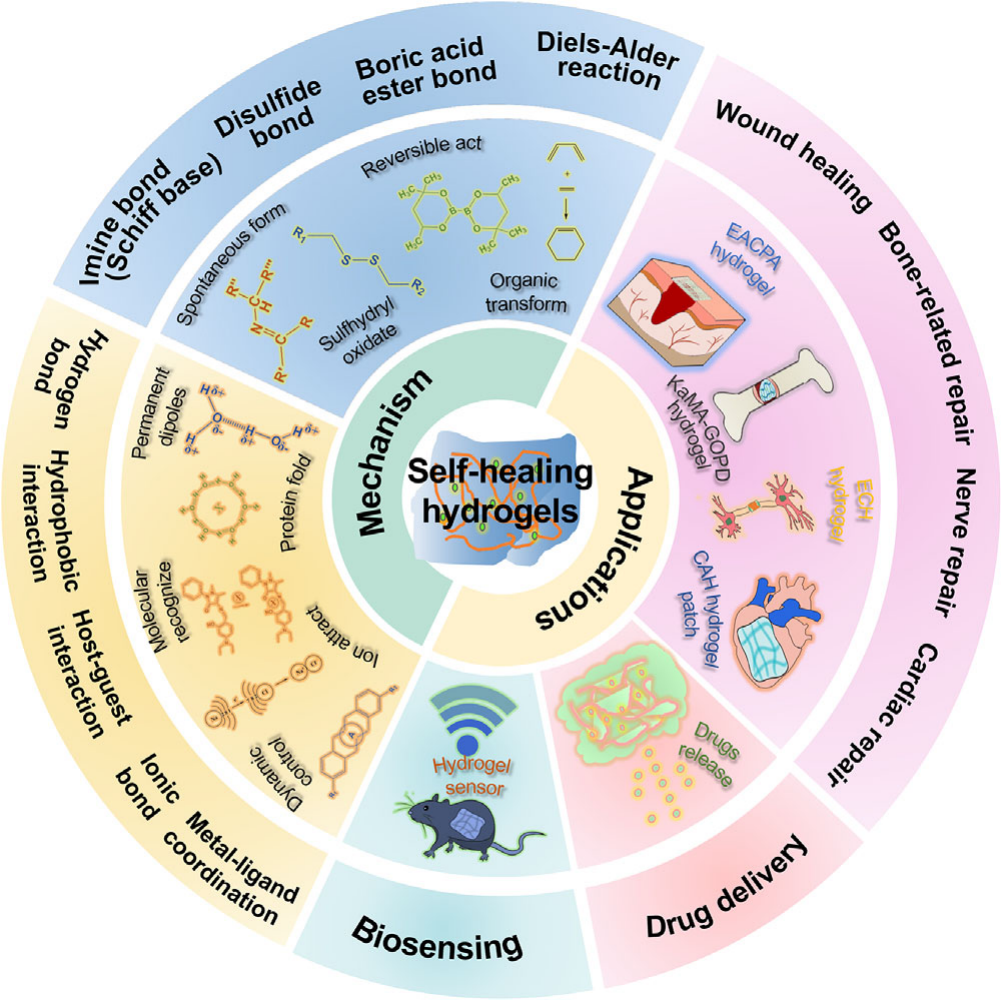
Self‐healing hydrogels: their mechanism and biological applications. (E‐A complex‐based polyacrylamide [EACPA] hydrogel; kappa‐carrageenan‐dopamine functionalized graphene oxide [KaMA‐GOPD] hydrogel; electroconductive [ECH] hydrogel; conductive and adhesive [CAH] hydrogel).

This review provides a comprehensive overview of self‐healing hydrogels, focusing on their classification, self‐repair mechanisms, and specific biomedical applications (Figure 1). Initially, we describe various types of natural hydrogels. Subsequently, we explore the mechanisms underlying the self‐healing properties of hydrogels, including dynamic covalent bonds, noncovalent interactions, and multimodal interactions. This review examines the use of self‐healing hydrogels across a broad spectrum of biomedical fields, including wound healing, bone repair, nerve regeneration, and cardiac tissue engineering. It concludes with an assessment of the challenges associated with the synthesis of these materials, their current limitations, and their potential to revolutionize healthcare and medical treatments in the future. In addition to tissue repair, self‐healing hydrogels offer innovative solutions for drug delivery, biosensing, and 3D bioprinting. With advances in research, these materials are expected to become cornerstones in the development of next‐generation biomedical technologies, addressing critical challenges in contemporary medicine.

## 2 Classification of Natural Self‐Healing Hydrogels

Following technological advancements, an increasing number of materials have been identified that are suitable for the formation of self‐healing hydrogels. Natural self‐healing hydrogels derived from biopolymers, such as polysaccharides and proteins, have been extensively studied for their potential biomedical applications. These hydrogels are particularly valuable owing to their biocompatibility, biodegradability, and tunable mechanical properties, which make them ideal candidates for tissue repair and regeneration. While composite hydrogels also hold considerable promise, this review focuses on natural hydrogels because of their unique advantages and the extensive research conducted in this area. Notably, polysaccharides and their derivatives—such as chitosan (CS) [27, 28, 29, 30], sodium alginate (SA) [31, 32], hyaluronic acid (HA) [33, 34, 35], cellulose [36, 37], k‐carrageenan [38, 39], agarose [40, 41], and glucomannan (GM) [42]—along with natural proteins like gelatin [43, 44], fibrin [45], and collagen [46, 47] have been extensively utilized. We provide a detailed overview of different natural hydrogels, aiming to highlight their specific performance and application advantages and their potential for biomedical applications (Table **1**).

**TABLE 1:** Types and characterizations of natural hydrogels (created by Chemdraw).

Types of natural hydrogels	Chemical formula and molecular formula	Chemical reaction	Characteristics	Mechanical significance	Measurement methods	Application	References
Chitosan (CS)	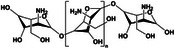 (C_6_H_11_NO_4_)*_n_*	Carboxylation reaction, acylation reaction, backbone hydrolysis	Antibacterial property	Biocompatibility and blood compatibility	Compression and tensile tests, colorimetric method	Drug carrier, film forming material, thickening agent, targeting preparation material	[27, 28, 29, 30]
Sodium alginate (SA)	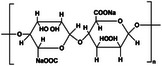 (C_6_H_7_NaO_6_)*_n_*	Proton‐catalyzed hydrolysis reaction, ion exchange reaction	Hygroscopicity, mobility	High water content and three‐dimensional network structure	Compression and tensile tests, viscosity measurement	Hemostatic, binding agent, thickening agent	[31, 32]
Hyaluronic acid (HA)	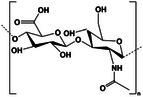 (C_14_H_21_NO_11_)*_n_*	Natural enzyme polymerization	Viscoelasticity, water retention	Lock in water and moisturize	Colorimetric and swelling methods	Treating arthritis, accelerate wound healing, protect the skin, a filler for joint surgery	[33, 34, 35]
Cellulose	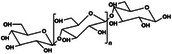 (C_6_H_10_O_5_)*_n_*	Hydrolysis reaction, combustion reaction	Hygroscopicity, swelling property	High strength, good toughness, and wear resistance	Particle size measurement and scanning electron microscopy	Dietary fiber, microcrystalline cellulose, pharmacy, papermaking, textile	[36, 37]
k‐Carrageenan	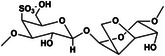 C_24_H_36_O_25_S_2_	React with protein, the hydroxyl group reacts chemically	Acid stability, gel property, solubleness, thickening property	Typical soft material properties	Sulfuric acid method and viscosity measurement	As common gel for jelly, detergent, cosmetics	[38, 39]
Agarose	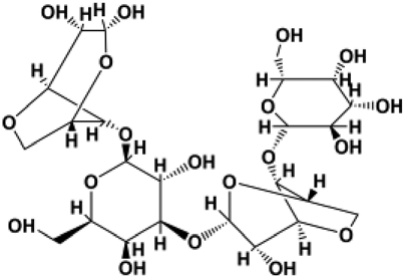 C_24_H_38_O_19_	Hydrolysis reaction	Water‐absorbing quality, biocompatibility	High mechanical strength and adsorption	Weight method and volume method	Agar fruit jelly, gel electrophoresis technique, isolation, and identification of nucleic acids	[40, 41]
Glucomannan (GM)	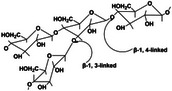 (C_6_H_10_O_5_)*_n_*	Esterification reaction, crosslinking, grafting	Water solubility, film‐forming property	Deacetylation and esterification modification	Spectrophotometry and performance liquid chromatography	Dietary fiber, gelatine	[42]
Gelatin	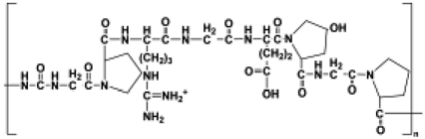 C_102_H_151_O_39_N_31_	Chromogenic reaction, electrostatic interaction	Surface activity, jelly strength, viscosity	Hydrophilicity, high viscosity, and transparency	Volume exclusion method and nuclear magnetic resonance method	Gelling agent, stabilizer, emulsifier, thickener, gelatin capsules	[43, 44]
Fibrin	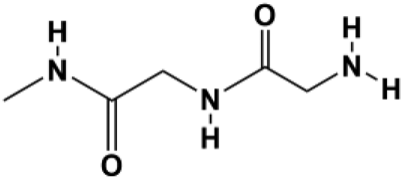 C_5_H_11_N_3_O_2_	Fibrinolysis (hydrolysis, sulfuric acid, and carboxylic acid)	Stability, mechanical strength	High elasticity and tension	Spectrophotometry and performance liquid chromatography	Basic support and external protective components of animal body	[45]
Collagen	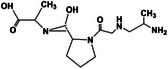 C_13_H_24_N_4_O_4_	Protease hydrolysis	Biocompatibility, biodegradability, bioactivity	High mechanical strength and flexibility	Ultraviolet spectroscopy and performance liquid chromatography	Biomedical material, human tissue, autologous skin graft	[46, 47]

### 2.1 Chitosan

CS, which is derived from the natural polysaccharide chitin via the removal of acetyl groups, is a cationic polyamine [48]. Glucosamine is the fundamental component of CS. Its inherent antibacterial properties and its diverse characteristics, such as biodegradability, biocompatibility, nontoxicity, moisture retention, and oxygen permeability, have made CS extensive applicable in biological contexts and contribute to pain relief in patients [49, 50]. Furthermore, owing to the abundant amino groups available, CS can function as a surface‐modifying agent and participate in crosslinking reactions [51].

As an illustration, Chen et al. [52] integrated a dodecyl‐modified CS (DCS) by modifying CS with dodecyl aldehyde. This unique structure, characterized by dodecyl tails, facilitates the insertion and anchorage of cell membranes, demonstrating remarkable tissue adhesion. Guo et al. [53] developed N‐carboxyethyl CS (CECS) by utilizing the Michael reaction in water without relying on organic solvents to address the solubility issues of CS, thus broadening considerably its applications. Liang et al. [54] created quaternized CS (QCS) via deacetylation of natural CS dissolved in organic acid. Zhao et al. [55] reported quaternized CS‐g‐polyaniline (QCSP) formed by grafting polyaniline onto QCS. These findings indicate that CECS, QCS, and QCSP exhibit superior water solubility and enhanced antibacterial properties compared with conventional CS. Additionally, Dong et al. [56] designed a CS‐grafted aniline tetramer by combining the amino groups of CS with the carboxyl groups of the aniline tetramer through an amidation reaction, endowing it with electroactive properties. Collectively, these surface modifications expanded considerably the application potential of CS.

### 2.2 Sodium Alginate

SA is a naturally occurring polymer derived from the interaction of iodine and mannitol obtained from brown algae such as Sargassum or kelp [57]. Its molecular structure is characterized by glycosidic linkages containing its chemical groups together [58]. SA exhibits notable biocompatibility, conductivity, and high viscosity [59]. Despite these advantageous properties, the mechanical properties of SA hydrogels are often limiting for its direct application.

Double network hydrogels were developed to address the limitations of the mechanical properties of SA hydrogels. For instance, Zhao et al. [60] formulated a physically crosslinked hydrogel by combining gelatin and SA and utilizing the crystalline domains of alginate acid for crosslinking. Building on this, Qiao et al. [61] further refined these hydrogels by incorporating tannic acid (TA), yielding novel hydrogels with high‐strain sensitivity. Zhao et al. [62] developed conductive composite hydrogels with multifunctional properties by incorporating poly (vinyl alcohol) (PVA), SA, and TA, and used borax as a cross‐linking agent to improve their effectiveness. Moreover, Chen et al. [63] created dopamine‐grafted oxidized SA, characterized by the presence of catechol groups in its chains, which enhanced considerably the hydrogel's properties. This underscores the versatility of SA and its derivatives in the development of advanced hydrogels with tailored properties for diverse biomedical applications.

### 2.3 Hyaluronic Acid

HA is a naturally occurring polysaccharide primarily obtained from animal tissues, chemical synthesis, and microbial fermentation [64]. It is extensively used as an injectable hydrogel dressing owing to its excellent biocompatibility, rapid gelation, appropriate biodegradability, and superior moisture retention [65]. HA plays a crucial role in wound healing by facilitating granulation, inflammation, and restoration of the epithelial layer. It provides a supportive matrix for fibroblasts and endothelial cells, thereby promoting their reorganization, proliferation, and migration during wound formation [66]. HA also enhances angiogenesis, thereby accelerating the wound‐healing process [67].

Despite its numerous benefits, HA has weak adhesive properties that may compromise the effectiveness of wound site closure. Studies have consistently shown that HA is essential for fetal wound healing, as fetal rabbit dermal fibroblasts express four times more HA receptors (including CD44) than adult dermal fibroblasts [68]. This high expression underscores the importance of HA in the rapid and scarless healing observed in fetal wounds. The high viscosity of HA poses challenges for applications, such as 3D printing. To address this limitation, Ouyang et al. [69] developed methacrylated HA, which serves as a component of multimaterial ink formulations for 3D printing. This modification enhances the processability of HA while maintaining its beneficial properties. Furthermore, Zhang et al. [70] created dopamine‐functionalized HA, which introduced catechol groups into the HA structure, thereby enhancing considerably its adhesiveness. This innovation not only improves the performance of HA in wound‐healing applications but also expands its potential for use in other biomedical contexts. Collectively, these advancements highlight the versatility and adaptability of HA for developing novel materials and expanding their applications in the biomedical field.

### 2.4 Cellulose

Cellulose, a naturally occurring polymer, is a versatile raw material that is used in various biomedical applications [71]. Polymeric cellulose synthesized using polyglucose as a crosslinking agent exhibits excellent biocompatibility and functions as an ideal antiadhesion material [72]. This characteristic helps mitigate postoperative complications caused by adhesions, thereby reducing the morbidity and mortality rates associated with surgical procedures.

In addition to its inherent properties, nanocellulose is extensively used in hydrogel development because of its excellent mechanical strength, biocompatibility, and modifiability. Zheng et al. [73] successfully synthesized (2,2,6,6‐tetramethylpiperidinooxy) TEMPO‐oxidized cellulose nanofibers (TOCNFs) through the TEMPO‐mediated oxidation of nanocellulose, thereby expanding the application potential of nanocellulose derivatives. Research has demonstrated that TOCNFs can form unique hierarchical structures that enhance the interactions between nanomaterials and hydrogel matrices. This structural attribute improves the crosslinking density, thereby contributing to a stable hydrogel network. Concurrently, Shao et al. [74] developed TA‐coated cellulose nanocrystals that exhibited outstanding adhesiveness owing to the presence of catechol groups in TA. These innovations highlight the potential of modified cellulose derivatives for enhancing the performance of hydrogels in various biomedical fields, including tissue engineering, wound healing, and antiadhesion strategies.

### 2.5 κ‐Carrageenan

κ‐Carrageenan, a hydrophilic colloid derived from red algae, is renowned for its ability to form gels that can be reversibly melted and solidified in the presence of potassium ions [75]. This unique characteristic enhances their appeal for a broad range of applications, particularly in the biomedical, food, and pharmaceutical sectors. C‐Phycocyanin (C‐PC), a dark blue pigment obtained primarily from Spirulina and prevalent in cyanobacteria, exhibits numerous beneficial properties, including antiallergic, antioxidant, and anticancer effects, as well as the ability to scavenge‐free radicals [76]. Furthermore, the notable red and near‐infrared (NIR) fluorescent properties of C‐PC make it an excellent fluorescent agent for in vivo imaging and monitoring applications [77, 78].

Given its antimicrobial, immunomodulatory, anti‐inflammatory, and antioxidant properties, which contribute to tissue regeneration and healing, C‐PC holds considerable potential for wound‐healing applications. Mihaila et al. [79] developed methacrylate κ‐carrageenan (KaMA), a photocrosslinkable version of κ‐carrageenan, which demonstrated improved mechanical properties and enhanced integrity. Furthermore, Guo's work [80] on ionically crosslinked κ‐carrageenan highlights the influence of cation addition on gelation. Both monovalent alkali ions and divalent cations can promote gelation by interacting with sulfate ester groups in κ‐carrageenan. These interactions enhance the formation of gel networks and contribute to the performance of materials in various fields, including drug delivery and tissue engineering. Overall, the combination of κ‐carrageenan and C‐PC presents promising avenues for developing advanced materials in biomedical applications, particularly for wound treatment and tissue regeneration.

### 2.6 Agarose

Agarose, a linear polysaccharide derived from agar, typically appears as white or yellow bead‐shaped gel particles or powder [81, 82]. It exhibits unique properties such as its ability to dissolve in water when heated at temperatures above 90°C and forms a semisolid hydrogel upon cooling to temperatures between 35 and 40°C [83]. Gelation occurs primarily through hydrogen bond formation, and alterations in these bonds can affect considerably the gelation process.

Owing to its neutrality and uncharged nature, agarose is extensively utilized in various applications, including biochemical analyses, clinical assays, and the extraction of biological macromolecules, such as deoxyribonucleic acid and proteins. These attributes make it a staple in both laboratory and research settings. Zhang et al. [84] advanced the use of agarose by designing hydrogels based on an agarose–ethylenediamine conjugate that exhibited self‐healing and injection capabilities. These properties enhance the versatility of agarose‐based materials and expand their suitability for biomedical applications. In particular, self‐healing provides a significant advantage for maintaining the integrity and performance of hydrogels in dynamic environments. Agarose continues to be valuable in both scientific and clinical contexts.

### 2.7 Glucomannan

GM is a macromolecular heteropolysaccharide composed of glucose and mannose residues linked by β‐1,4‐glycosidic bonds [42, 85]. This unique structure endows GM with several valuable chemical properties, including high‐water solubility, reversibility, film‐forming ability, and thickening capabilities. These properties render GM suitable for a broad range of applications in food, pharmaceutical, and biomedical industries.

One of the most notable biomedical functions of GM is its ability to bind specifically to mannose receptor ligands on macrophages. This interaction boosts the effectiveness of the hydrogel in guiding polarization toward the M2‐like macrophage phenotype, which is linked to the processes of wound healing and tissue regeneration. By encouraging M2 macrophage polarization, the use of GM‐infused hydrogels notably enhanced the rate of which wounds heal [86]. Wei et al. [87] developed hydrogels based on oxidized konjac GM (OKGM). Oxidation enhances the properties of GM and exerts anti‐inflammatory effects by activating macrophages. This advancement provides potential therapeutic benefits for managing inflammation and enhancing the effectiveness of hydrogels in wound care applications. Overall, the use of GM and its derivatives in hydrogels is an encouraging strategy for the production of efficient materials for tissue engineering and regenerative medicine.

### 2.8 Gelatin

Gelatin is a hydrophilic macromolecular colloid that originates from the partial hydrolysis of collagen, a structural protein found in connective tissues [43, 88]. As a protein macromolecule, gelatin exhibits characteristics typical of proteins. However, its unique molecular structure imparts distinctive physical and chemical properties [89]. Notably, gelatin is insoluble in cold water; however, when immersed in cold water it can absorb 5–10 times its weight in water, leading to swelling and softening. Upon heating, gelatin dissolves into a colloidal solution; additionally, when cooled to temperatures below 35–40°C, it forms a gel [90, 91]. This reversible gelation process is key to its application in various fields, including food science, pharmaceuticals, and tissue engineering.

Commercially, gelatin is primarily sourced from animal by‐products, including skin, leather waste, and bones. Their widespread availability has contributed to their use in many industrial applications. Yue et al. [92] explored the synthesis of gelatin‐based hydrogels with integrated conductivity and self‐healing capabilities. They used a technique that involved bonding methacrylic anhydride (MA) to gelatin to create double‐bond functionalized gelatin. This modification not only enhances the mechanical properties of the hydrogel but also opens avenues for applications where electrical conductivity and self‐repairing features are essential, such as in bioelectronics or smart materials. The ability of gelatin to function as a hydrogel precursor demonstrates its potential for innovative applications in biomedicine, including wound healing, tissue repair, and drug delivery.

### 2.9 Fibrin

Fibrin, commonly referred to as blood fibrin, is a highly insoluble protein polymer that plays a crucial role in the body's hemostatic response [45, 93]. It is typically formed by the conversion of fibrinogen, a soluble plasma protein, under physiological conditions [94]. The resulting fibrin structure, resembling fine needles or fibers, creates a meshwork that acts as a scaffold at the site of the injury. Fibrin, which is primarily derived from plasma proteins, exhibits excellent biocompatibility and low toxicity, making it an ideal material for various clinical applications [95]. These properties have led to its extensive use as a hemostatic agent to control bleeding in surgical procedures and traumatic injuries, as well as a wound‐healing agent because of its capacity to enhance tissue repair.

Fibrin improves considerably wound healing by forming a scaffold that aids cell adhesion and migration, both of which are essential for tissue regeneration [96]. The porous nature of fibrin allows cells, including fibroblasts and endothelial cells, to infiltrate and support the formation of new tissues. Moreover, studies have demonstrated that fibrin possesses bactericidal properties that can help reduce the risk of wound infection [97]. As a biodegradable biomaterial, fibrin breaks down naturally in the body once its therapeutic function is completed, thereby eliminating the need for surgical removal [98]. This combination of features has led to the ongoing application of fibrin in clinical environments for wound care, tissue engineering, and regenerative medicine. Overall, the multifunctionality and compatibility of fibrin with biological systems render it a valuable component for various biomedical applications.

### 2.10 Collagen

Collagen, the most abundant protein in mammals, enhances the mechanical strength of various tissues, including skin, bones, and cartilage [47]. Its unique triple‐helical structure, which is conserved across species, enables it to maintain its biological activity, making it a focal point for biomedical applications and extensive research [99].

To create hydrogels, researchers have developed methacrylamide‐modified collagen, which enables hydrogel production via the ultraviolet (UV)‐triggered radical polymerization of methacrylate components [100]. Although this strategy can create valuable hydrogels, practical applications may be limited by factors such as the level of collagen modification, concentration of photo‐initiators, and dosage of UV light exposure. A major advancement was made by Pupkaite et al. [101] who introduced a method to modify collagen to incorporate thiol groups, thus enabling the formation of injectable hydrogels through crosslinking via a Michael addition reaction. This method provides enhanced control over the properties of the resulting hydrogels by altering their preparation parameters. Most notably, these hydrogels exhibit shear‐thinning and self‐healing characteristics, allowing easy injection into the gel state. This feature is particularly advantageous for biomedical applications in which minimally invasive delivery is crucial, including tissue engineering and regenerative medicine.

## 3 Self‐Healing Mechanisms

Self‐healing hydrogels exhibit remarkable potential for application in tissue engineering and biomedicine because of their high strength, self‐healing capabilities, and viscoelastic properties [102]. Various techniques have been employed to evaluate the mechanical properties and self‐healing capacities of these hydrogels, including tensile, shear, and dynamic mechanical analysis [103, 104, 105]. Specifically, tensile testing quantifies the elasticity and toughness of hydrogels, whereas shear testing assesses their resistance to deformation. Dynamic mechanical analysis provided insights into the viscoelastic characteristics and recovery mechanisms of hydrogels. Additionally, their microstructures and compositions have been studied using scanning electron microscopy and nuclear magnetic resonance [106, 107]. These testing methods help clarify the applicability of hydrogels in diverse scenarios. For example, the restoration of mechanical strength can be quantified by measuring the tensile strength and elongation at break before and after the healing process. The kinetics of the healing process can be elucidated by monitoring the time‐dependent recovery of mechanical properties such as the storage modulus (*G'*) and loss modulus (*G*’) using dynamic mechanical analysis.

Self‐healing hydrogels exhibit unique mechanical properties that are characterized by their elasticity, toughness, and recovery mechanisms. These properties are crucial for applications in biomedicine and tissue engineering. The self‐healing function of these hydrogels is attributed to the interaction of dynamic covalent bonds [108, 109, 110] and noncovalent bonds [111, 112], resulting in excellent mechanical performance and outstanding biocompatibility [113, 114]. Dynamic covalent bonds were initially identified and encompassed imine bonds (Schiff bases) [115], disulfides [116, 117], boric acid esters [118, 119], and Diels–Alder reactions [120, 121]. Noncovalent interactions include hydrogen bonding [122], hydrophobic interactions [123], ionic bond [124], host–guest interactions [125], and metal–ligand coordination [126]. Multimodal interactions can occur when dynamic covalent bonds are coupled with noncovalent interactions or when two or more components are crosslinked. Self‐healing hydrogels have been successfully fabricated using advanced techniques. Herein, we introduce the mechanisms of self‐healing hydrogels based on dynamic covalent bonds and noncovalent interactions, focusing on their properties and potential applications (Table 2). In future, we aim to introduce novel concepts for the development of sophisticated self‐healing hydrogels.

**TABLE 2:** Mechanisms of dynamic covalent bonds and noncovalent interactions of self‐healing hydrogels (created by Chemdraw).

Types of mechanisms	Specific classification and self‐healing mechanism	Reaction mechanism	Characteristics	Application	References
Dynamic covalent bonds	Imine bond (Schiff base) 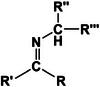	Through the condensation reaction of amines and aldehydes	Bacteriostatic, bactericidal, antitumor, antiviral	Catalyst, organic synthesis reagents, liquid crystal materials	[115]
	Disulfide bond 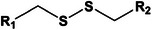	Formed by the coupling of two thiol groups	Stability, dynamic change	Maintain the molecular structure of proteins	[116, 117]
	Boric acid ester bond 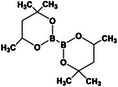	Formed by the reaction of boric acid with alcohol	Good activity, reversible and dynamic	Applied to the study of enzyme reaction mechanism	[118]
	Diels–Alder reaction 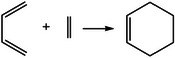	Cycloaddition reaction	Rich stereochemical presentation	Synthesis of polycarbon rings and polyheterocyclic compounds ‌	[120, 121]
Noncovalent interactions	Hydrogen bond 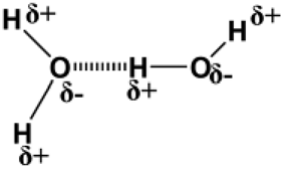	Intramolecular interaction	Directivity, saturability	The stability of the compound is affected	[122]
	Hydrophobic interaction 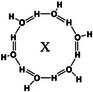	Hydrophobic group	Hydrophobicity	Maintain protein conformation	[123]
	Host–guest interaction 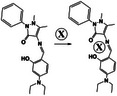	Physical crosslinking	Stimulus responsiveness	Self‐healing	[124]
	Ionic bond 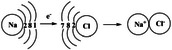	Anions or cations are formed by electrostatic interaction	Strong ‌ electric interaction	Synthesis of ionic compounds, ion exchange reactions	[125]
	Metal–ligand coordination 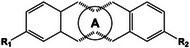	Formed by a coordination bond	Stability, solubility	Catalyst, transition metal complexes, or salts	[126]

### 3.1 Dynamic Covalent Bonds

Dynamic covalent bonds play a crucial role in the self‐healing of hydrogels. These bonds can reversibly form and break, allowing the hydrogel network to reorganize and restore its integrity after damage. The self‐healing process was quantified by measuring the tensile strength and elongation at break before and after the healing process. For example, hydrogels containing imine bonds (Schiff bases) can undergo spontaneous self‐healing owing to the dynamic nature of these bonds. Similarly, disulfide bonds, boric acid ester bonds, and Diels–Alder reactions contribute to the self‐healing properties of hydrogels by providing reversible crosslinking sites.

#### 3.1.1 Imine Bond (Schiff Base)

The imine bond, which results from the condensation reaction between an amine and an aldehyde group, forms a carbon–nitrogen double bond and is classified as a dynamic covalent bond, alternatively known as a Schiff base [127]. This bond is lauded for its inherent self‐healing characteristics within hydrogel systems, which facilitates frequent breaking and regeneration, thereby conferring substantial application potential [115]. Notably, imine bonds are among the few dynamic covalent reactions capable of spontaneous formation, which have led to their extensive application in dynamic covalent hydrogels.

The self‐repairing properties of hydrogels are illustrated by the formation of imine bonds between the amino groups of DCS and the aldehyde groups in a four‐armed polyethylene glycol terminated with benzaldehyde (BAPEG) [128]. Macroscopic self‐healing demonstrations were conducted on DCS/BAPEG hydrogels, demonstrating the ability of the two pregelled butterfly shaped hybrid hydrogels to coalesce into an intact structure without external forces. These self‐healing hydrogels exhibited remarkable stability and were sufficient to support the weight. Furthermore, the oxidation of GM by sodium periodate yields GM‐aldehyde (GM‐CHO), which forms imine bonds when combined with the Q11 (Ac‐QQKFQFQFEQQ‐Am) peptide in solution. It has been observed that these hydrogels, while susceptible to breaking under duress, rapidly self‐heal upon the removal of stress, thereby enhancing their stability and self‐healing attributes.

#### 3.1.2 Disulfide Bonds

A disulfide bond is a stable covalent link formed by the oxidation of sulfhydryl groups from separate cysteine residues, which can occur either within a single peptide chain or between multiple chains [116]. It plays a pivotal role in protein architecture by ensuring the correct folding and stability of peptide chains. There is a direct correlation between the number of disulfide bonds and structural integrity of the protein. These bonds are instrumental in sculpting the three‐dimensional structure of proteins and are particularly amenable to the formation of dynamic covalent hydrogels [129].

A unique characteristic of disulfide bonds is their susceptibility to reduction by reducing agents [130]. They are robust and capable of forming at low temperatures, but are readily cleavable upon exposure to photo‐irradiation or thermal stress. In hydrogel design, sulfur chemistry has been employed to fabricate redox‐sensitive sol–gel systems. Thiols can react with disulfide moieties, resulting in the formation of a disulfide and thiol, thus allowing crosslinking into a stable three‐dimensional network [131]. Moreover, they serve as effective crosslinking agents, providing a rich array of active sites for further functionalization [117]. Consequently, extensive research has been directed toward systems based on disulfide bonds. The dynamic covalent crosslinking afforded by these bonds has emerged as a potent strategy for the development of self‐healing hydrogels. Hydrogels are at the forefront of the biomedical field and offer a promising avenue for the advancement of biomaterials and therapeutics.

#### 3.1.3 Boric Acid Ester Bond

Boric acid ester bonds, which are dynamic covalent bonds, can be effectively employed to prepare self‐healing hydrogels [118]. These bonds are formed through reversible interactions between boric acid and the diols. Extensive research has demonstrated the utility of boronic acids and their derivatives in the design of self‐healing hydrogels, such as phenylboronic acid (PBA) and polymers incorporating PBA.

For instance, the catechol groups in epigallocatechin‐3‐gallate (EGCG) can interact with the boric acid of PBA to form two reversible boric acid ester bonds, acting as dynamic crosslinkers in advanced hydrogels [132]. These hydrogels exhibit moderate tissue adhesion, facilitating their application and allowing easy removal from the skin without any residue. Notably, the two hydrogel pieces self‐healed efficiently, regaining their structural integrity after just 3 min of contact. In addition, 2‐formylphenylboronic acid (2‐FPBA) can react with cis‐diols, resulting in the formation of reversible boric acid esters that respond to variations in pH and the presence of saccharides such as glucose [133]. Typically, 2‐FPBA crosslinked hydrogels are developed under alkaline conditions, potentially limiting their applications in the biomedical field.

#### 3.1.4 Diels–Alder Reaction

The Diels–Alder reaction is a pivotal organic transformation in which a conjugated diene engages with an olefin or alkyne to form a cyclohexene or 1,4‐cyclohexadiene ring system [120]. This synthetic strategy is not limited to carbon atoms and can be extended to include other elements within the newly formed ring. This reaction is highly valued in modern organic synthesis because of its efficiency, selectivity, and yield [134]. In the domain of self‐healing hydrogels, the Diels–Alder reaction has been used to fabricate materials capable of autonomous repair.

Researchers have harnessed furyl/maleimide pairs to develop an innovative self‐healing hydrogel through a reversible Diels–Alder reaction, with cellulose nanocrystals functioning as both a strengthening component and chemical crosslinker [135]. By modifying the degree of substitution of the furyl functional groups, molar ratio of furyl to maleimide in the gelation process, and the swelling ratio of the gels, the mechanical properties and self‐healing capabilities of these hydrogels can be finely tuned. Moreover, hybrid hydrogels produced by Diels–Alder reactions between furan‐modified pectin and maleimide‐modified CS exhibit remarkable self‐healing properties, even under mild conditions [136]. These hydrogels could potentially withstand a load of 500 g without incurring damage, demonstrating their potential for applications requiring both self‐healing and mechanical robustness.

### 3.2 Noncovalent Interactions

Noncovalent interactions, such as hydrogen bonding, hydrophobic interactions, host–guest interactions, ionic bonds, and metal–ligand coordination, also contribute considerably to the self‐healing ability of hydrogels. These interactions are dynamic, and can be reformed after disruption, allowing the hydrogel to recover its structure and function. For instance, hydrogen bonds between the polymer chains can break and reform under stress, thereby enabling the hydrogel to self‐heal. The kinetics of the healing process can be elucidated by monitoring the time‐dependent recovery of mechanical properties such as the storage modulus (*G*’) and loss modulus (*G*’) using dynamic mechanical analysis.

#### 3.2.1 Hydrogen Bond

Hydrogen bonding, a type of intermolecular force, arises from permanent dipoles formed when a hydrogen atom covalently linked to a highly electronegative atom interacts with another electronegative atom [137, 138]. This interaction is pivotal for the formation of self‐healing hydrogels, particularly within cationic guar gum, which possesses an abundance of hydroxyl groups and branches, capable of engaging in strong intermolecular hydrogen bonds. A rapid‐forming, injectable, self‐healing, and conductive slime was engineered using cationic guar gum, demonstrating its utility in wound healing and tissue regeneration within a minute [139]. The self‐healing attribute of this hydrogel system is facilitated by the dynamic nature of hydrogen bonds, which allows for the reformation of the hydrogel network following damage, thereby enhancing its stability and therapeutic potential.

#### 3.2.2 Hydrophobic Interactions

Hydrophobic interactions are pivotal for protein folding and represent an intermolecular force arising from permanent dipoles [123]. These interactions occur when hydrophobic groups congregate to avoid contact with water, a phenomenon known as hydrophobic interactions. These interactions are crucial for maintaining protein conformation because water molecules exhibit stronger interactions with themselves than with nonpolar molecules [140]. The crosslinking mechanisms in hydrophobic interactions involve engagements among water and the hydrophobic groups of hydrophobic substances, which are typically nonpolar groups. This interaction promoted the aggregation of hydrophobic groups and intensified the structuring and concentration of water.

**FIGURE 2:**
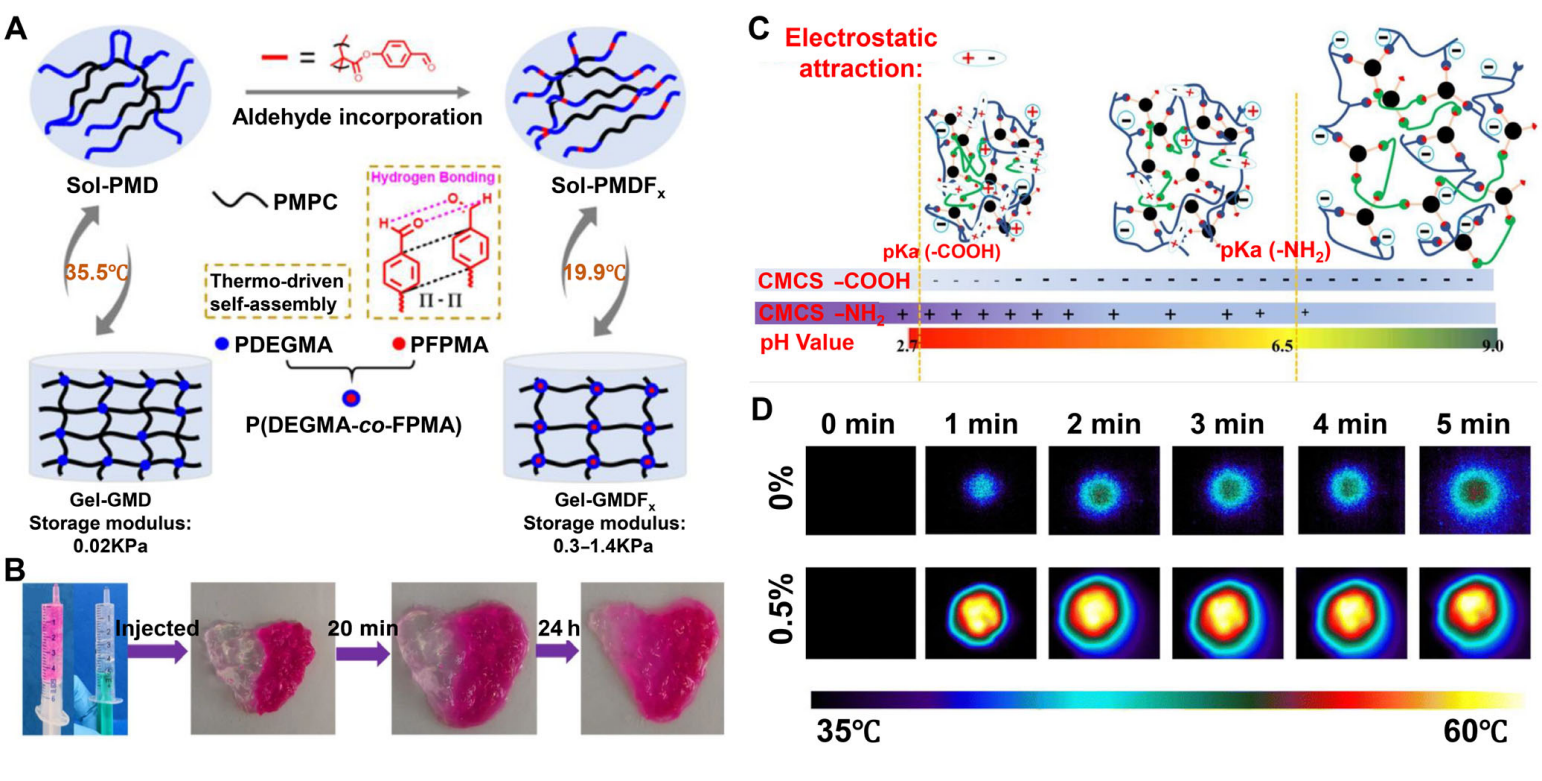
Various crosslinked self‐healing hydrogels. (A) Preparation process of temperature‐responsive aldehyde hydrogel [22], Copyright©2022, American Chemical Society. (B) Self‐healing macro diagram of hydrogel sample at pH = 7. (C) Swelling behavior diagram of hydrogel at different pH values [23], Copyright©2020, Elsevier. (D) Temperature of different hydrogels under different time of laser irradiation [21], Copyright©2024, Royal Society of Chemistry.

By grafting tert‐butyl pyridine onto the chains of bromobutyl rubber, researchers developed micelle‐like structures that formed a hydrophobic layer, safeguarding the ionic bonds of ionomers [27]. Consequently, the ionomer exhibits exceptional resistance to water, acids, and alkalis and can completely self‐heal under adverse environmental conditions. This innovation underscores the potential of hydrophobic interactions in the development of materials with self‐healing capabilities, particularly in adverse environments. As Zhao et al. [22] discovered, the use of hydrophobic interactions among thermoresponsive polymer segments, physical interactions of benzaldehyde groups, and physical crosslinking can yield an injectable thermoresponsive self‐healing hydrogel (Figure 2A), which provides favorable assistance for biomedical applications. Yu et al. [23] designed a novel self‐healing hydrogel that was crosslinked by imine bonds and exhibited excellent pH‐sensitive swelling properties. When the transparent hydrogel and hydrogel containing red rhodamine B dye were placed in a buffer solution (pH = 7), subsequently injected into a Petri dish using a syringe, and incubated for 20 min, it was found that the two hydrogels integrated together (Figure 2B), demonstrating the potential of this hydrogel for self‐healing. Additionally, electrostatic attraction at different pH values caused the hydrogel to undergo contraction and expansion (Figure 2C). Rybak et al. [21] developed an antibacterial composite thermosensitive self‐healing hydrogel, which could effectively heat up to 60°C under laser irradiation conditions to exert antibacterial effects. The temperature images of the hydrogels at different times before laser irradiation are shown in Figure 2D. In summary, the unique structure of hydrogels enables them to exert fully their biomedical functions under light, heat, and pH conditions.

#### 3.2.3 Host–Guest Interactions

Host–guest interactions facilitate physical crosslinking through the simultaneous recognition of host and guest components at the molecular level, with the complex interplay between guest molecules and macrocyclic host molecules [125]. This unique structure limits the extent of crosslinking while maintaining the strength and self‐healing properties. In recent years, the cyclodextrin (CD) family has been selected for the design of self‐healing hydrogels via host–guest interactions. Researchers have created self‐repairing conductive hydrogels that incorporate β‐cyclodextrin (β‐CD), multiwalled carbon nanotubes (CNT), N‐isopropylacrylamide (NIPAM), and nanostructured poly‐pyrrole (PPY) [141]. In this fabrication process, β‐CD serves as the host molecule, NIPAM serves as the guest molecule, PPY serves as the highly conductive component, and CNT acts the physical crosslinker and conducting substrate. These novel hydrogels exhibit high conductivity, flexibility, have stable and elastic mechanical properties, possess excellent self‐healing properties and satisfactory stimuli‐responsiveness, and are outstanding candidates for applications in electrical devices that respond to stimuli and in artificial organs. Additionally, researchers have reported the development of supramolecular materials based on poly (acrylic acid) (pAA) modified with CDs (pAA‐CDs) as a host polymer and pAA functionalized with ferrocene (pAA‐Fc) as a guest polymer, demonstrating self‐healing capabilities through host–guest interactions [142]. Furthermore, a change in the redox potential can result in a reversible solution‐to‐hydrogel state transition. Given their satisfactory stimulus responsiveness and excellent self‐healing properties, the hydrogels presented herein possess undeniable potential as stimuli‐responsive carriers for drug delivery and materials for peripheral vascular embolization, specifically targeting cancer cells and myomas.

#### 3.2.4 Ionic Bonds

Ionic bonds, also known as salt bonds, are created when two or more atoms or chemical groups loose or gain electrons to form ions [124]. These ions interact through electrostatic forces owing to their opposite charges. The attraction between oppositely charged ions leads to the formation of ionic bonds that are comparable in strength to covalent bonds.

Reversible ionic bonds, such as those formed between polyethylenimine‐functionalized silicon nanoparticles (PEI‐SiNPs) and HA in a nanocomposite hydrogel, provide the dynamic network properties necessary for shear‐thinning and self‐healing behavior [143]. The presence of excess charged amines in PEI‐SiNPs, in addition to their role in gelation, enhanced the loading and subsequent release of anionic anticancer drugs, such as methotrexate, after the formation of the hydrogel. This highlighted the utility of ionic interactions in the design of responsive and therapeutic hydrogel systems.

#### 3.2.5 Metal–Ligand Coordination

Metal–ligand coordination interactions can regulate the properties of materials by changing the metal ions, counter‐ions, and ligands. Therefore, the regulation of its dynamic properties has become an important research direction in materials science [144]. These interactions have been effectively utilized to engineer self‐healing hydrogels. For instance, coordination between iron and bisphenol ligands has been shown to produce substances with self‐healing properties.

Shi et al. [145] utilized metal–ligand dynamic coordination bonds to fabricate a moldable hydrogel based on HA, which was modified with pendant bisphosphonate ligands and crosslinked reversibly. The hydrogel prepared by introducing silver ions into an HA solution containing bisphosphonate groups exhibited exceptional properties for clinical applications, including the ability to conform to irregularly shaped wound defects without requiring remodeling. Additionally, the hydrogel demonstrated antimicrobial activity against both Gram‐positive and Gram‐negative bacteria, facilitating infection prevention during wound treatment. In vivo evaluation of rat skin wounds indicated a considerable increase in skin layer regeneration without residual wounds [146]. This “ready‐to‐use” hydrogel demonstrates self‐healing and malleable properties, making it the best choice for repairing wounds.

Rowan et al. [147] reported photoactivated healable supramolecular polymers that utilize the coordination between La^3+^ and Zn^2+^ metal ions as well as 2,6‐bis(1‐methylbenzimidazolyl) pyridine for enhanced functionality. Holten et al. [148] developed self‐healing gels incorporating Fe^3+^–catechol coordinate bonds to enhance their healing properties. Guan et al. [149] created a hard/soft two‐phase brush copolymer that exhibited strong self‐healing ability at room temperature owing to the addition of Zn^2+^–imidazole interactions to the soft matrix. Rao et al. [150] designed a hydrogel with excellent stretchability and self‐healing ability by embedding a two‐strength dynamic metal–ligand coordination bond. These advances highlight the versatility of metal coordination interactions in the development of customized self‐healing hydrogels for a variety of biomedical applications, including wound care and drug delivery.

### 3.3 Multimodal Interactions

Our research has demonstrated that dynamic covalent bonds and noncovalent interactions can function independently within self‐healing hydrogels to achieve rapid and effective repair. However, in practical applications, these two types of bonds can be combined to leverage the strengths of both the covalent and noncovalent bonds. This combination enhanced the mechanical strength of the hydrogel and accelerated the healing rate [151]. For example, injectable self‐healing hydrogels exhibit rapid gelation and cysteine‐specific on‐demand dissolution responses [113]. This hydrogel is based on a series of dynamic covalent bonds formed via the Knoevenagel condensation reaction and boronate linkages, which produce C = C double bonds. These bonds confer excellent injectability and self‐healing ability to the hydrogel.

Physical hydrogels are ideal candidates for functional and biomaterial applications owing to their unique properties. However, their poor mechanical properties often limit their practical applications [152, 153]. To address this issue, researchers have developed robust physical hydrogels based on multimodal interactions crosslinking [154]. This hydrogel uses HA as a template and incorporates graphene oxide (GO) and laponite clay sheets into the gel system [155]. The surfactants in the HA system acted as mutual stabilizers between the two nanoplates, allowing for the addition of higher concentrations of nanoplates to the gel network. This enhances the overall mechanical properties of the hydrogels. Consequently, physical hydrogels exhibit ideal mechanical properties, including high strength and elasticity, self‐healing ability, and shape‐memory ability. These properties were achieved through hydrophobic bonding domains and crosslinking between the two nanoplates.

In another study, researchers used to design self‐healing hydrogels. However, the relatively slow dissolution of cationic GG in water at different concentrations limits its processing and applications [139]. Therefore, researchers have adopted new methods to solve this problem. They alternately added acids and bases to form hydrogen bonds between hydrogen ions and hydroxyl groups, and then added bases to neutralize excess hydrogen ions [156]. Building on this method, they selected a commercially available acidic poly(3,4‐ethylenedioxythiophene): polystyrene sulfonate (PEDOT: PSS) solution to prepare a conductive PEDOT: PSS/guar gum (PPGS) hydrogel. PEDOT: PSS, which contains many negatively charged groups, interacts electrostatically with the side chains of the cationic guar gum. The resulting gel had physiological pH and exhibited excellent self‐healing properties.

## 4 Biomedical Applications

### 4.1 Tissue Repair

Despite the plethora of commercial products available for wound healing, bone repair, nerve regeneration, and cardiac repair, these products are often prohibitively expensive and disposable [157]. Moreover, these commercial materials are frequently incompatible with human physiology, posing a risk of secondary injury during removal or replacement [158]. To address these issues, self‐healing hydrogels with distinctive structures and properties have emerged. Self‐healing hydrogels are poised to surpass conventional products in terms of reliability, and are considered promising candidates for a range of biomedical applications [159]. However, despite a range of approaches for generating self‐healing hydrogels, their clinical applications remain limited. Through a combination of physical and chemical strategies, self‐healing hydrogels can be endowed with injectable antibacterial properties, electrical conductivity, and so on. The self‐healing efficiency was quantified by measuring the tensile strength and elongation at break before and after the healing process, which demonstrated the rapid recovery of the mechanical properties. These findings highlighted the potential of self‐healing hydrogels in wound care, osseous repair, and neural regeneration [160].

#### 4.1.1 Wound Healing

**FIGURE 3:**
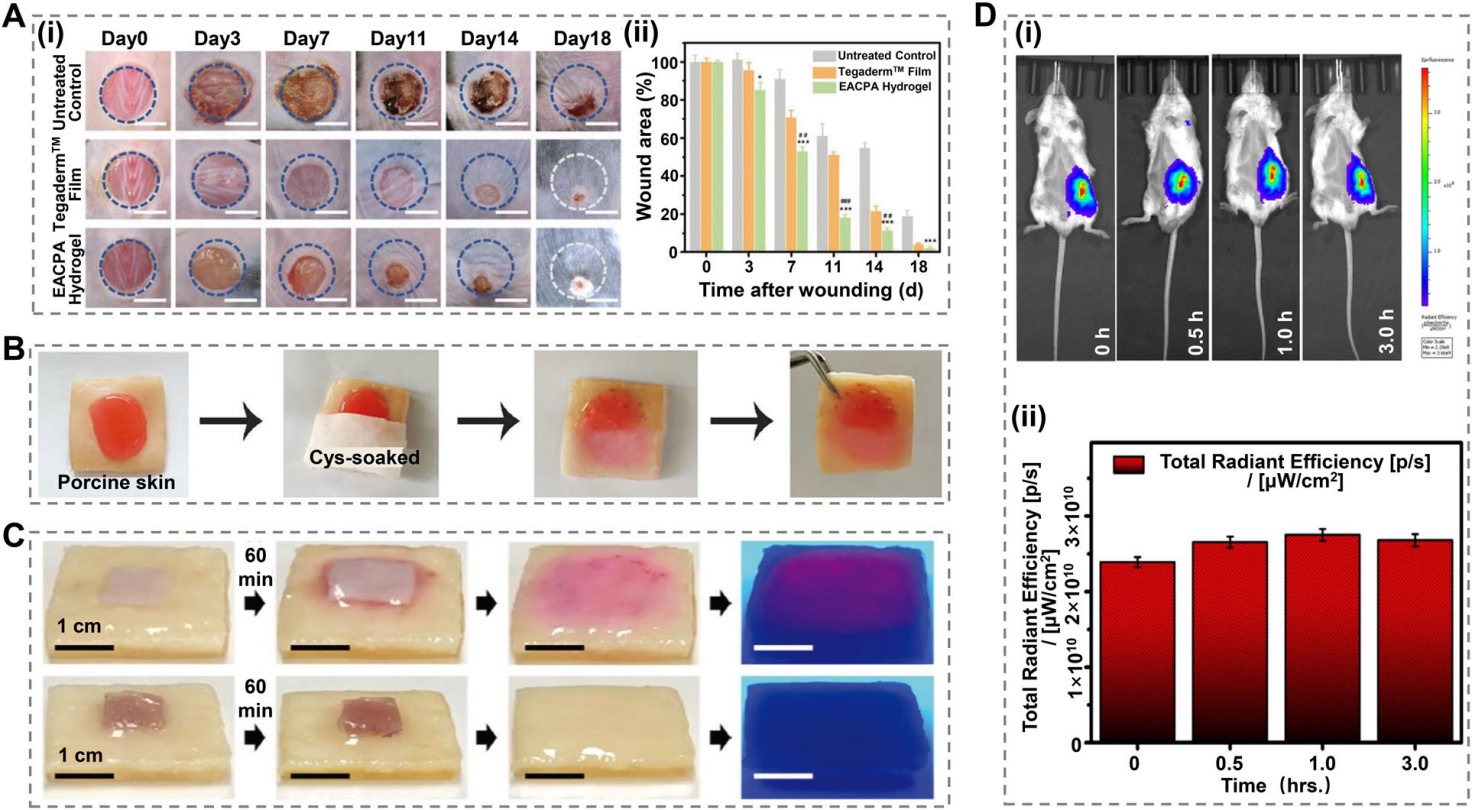
Mechanisms by which various hydrogels facilitate wound healing. (A) (i) Photographs of diabetic chronic wounds treated with different interventions. (ii) Quantification of wound closure rates [163], Copyright©2021, John Wiley and Sons. (B) Tissue image of 2‐FPBA crosslinked hydrogel applied to porcine skin [133], Copyright©2021, John Wiley and Sons. (C) Demonstration of Nile Red model cargo release onto porcine skin [164], Copyright©2020, John Wiley and Sons. (D) (i) In vivo imaging of hydrogels at different time points. (ii) The total radiation efficiency of hydrogels was measured at different time intervals [165], Copyright©2020, Elsevier.

Wound healing is a complex process. In response to external trauma, the skin reacts immediately and adapts to various wound sizes. A larger wound area typically requires more time for recovery and may result in permanent damage despite meticulous care [161, 162]. Traditionally, self‐healing hydrogels have been used to address these challenges. Zhao et al. [163] developed a novel E‐A complex‐based polyacrylamide (EACPA hydrogel) by incorporating EGCG, 3‐acrylamido phenylboronic acid (APBA), and acrylamide, which demonstrated excellent mechanical properties, self‐healing capabilities, and tissue adhesiveness characteristics. The advantage of this hydrogel is that the catechol groups in EGCG form borate bonds with APBA, allowing it to exist in a dynamically crosslinked form. By conducting a comparative analysis among the EACPA hydrogels, commercial Tegaderm dressing, and nontreated groups over an 18‐d period, it was determined that the EACPA hydrogel exhibited superior efficacy in wound healing (Figure 3A(i)). In addition, the wound area was assessed during the experimental period using photographs. Furthermore, the gradual release of EGCG from the hydrogel aided its dissolution, thus mitigating the risk of secondary injury (Figure 3A(ii)).

Furthermore, hydrogels can achieve self‐healing properties through dynamic covalent or noncovalent bond interactions, and appropriate physical modifications can facilitate the rapid release of drug payloads, thereby expediting tissue healing. Ding et al. [133] developed a novel hydrogel (2‐FPBA crosslinked hydrogel) capable of controlled dissolution by combining 2‐FPBA with cyanoacetate end‐functionalized 4‐arm polyethylene glycol and PVA. This hydrogel was characterized by the preparation of a series of dynamic covalent bonds constructed by the Knoevenagel condensation reaction and the C = C double bond produced by the borate ester bond. Because of the presence of a thiazolidino boronic acid complex, these hydrogels dissolved rapidly in the presence of cysteine (Figure 3B). Shin et al. [164] reported a hydrogel synthesized by mixing PVA and poly (hydroxyethyl methacrylate) micelles and demonstrated the release of a model drug. Specifically, Nile red, as a model cargo, was retained in the H0‐gel while being released rapidly from the H43‐gel, as evidenced by optical microscopy observations (Figure 3C). Dev et al. [165] created a hydrogel utilizing κ‐carrageenan and PC, which exhibited in vivo fluorescence imaging capabilities. The far‐red and NIR imaging properties indicated that radiant efficiency remained stable over 0.25–3 h, highlighting the potential for the wound treatment process to be monitored and located at any time (Figure 3D(i‐ii)).

**FIGURE 4:**
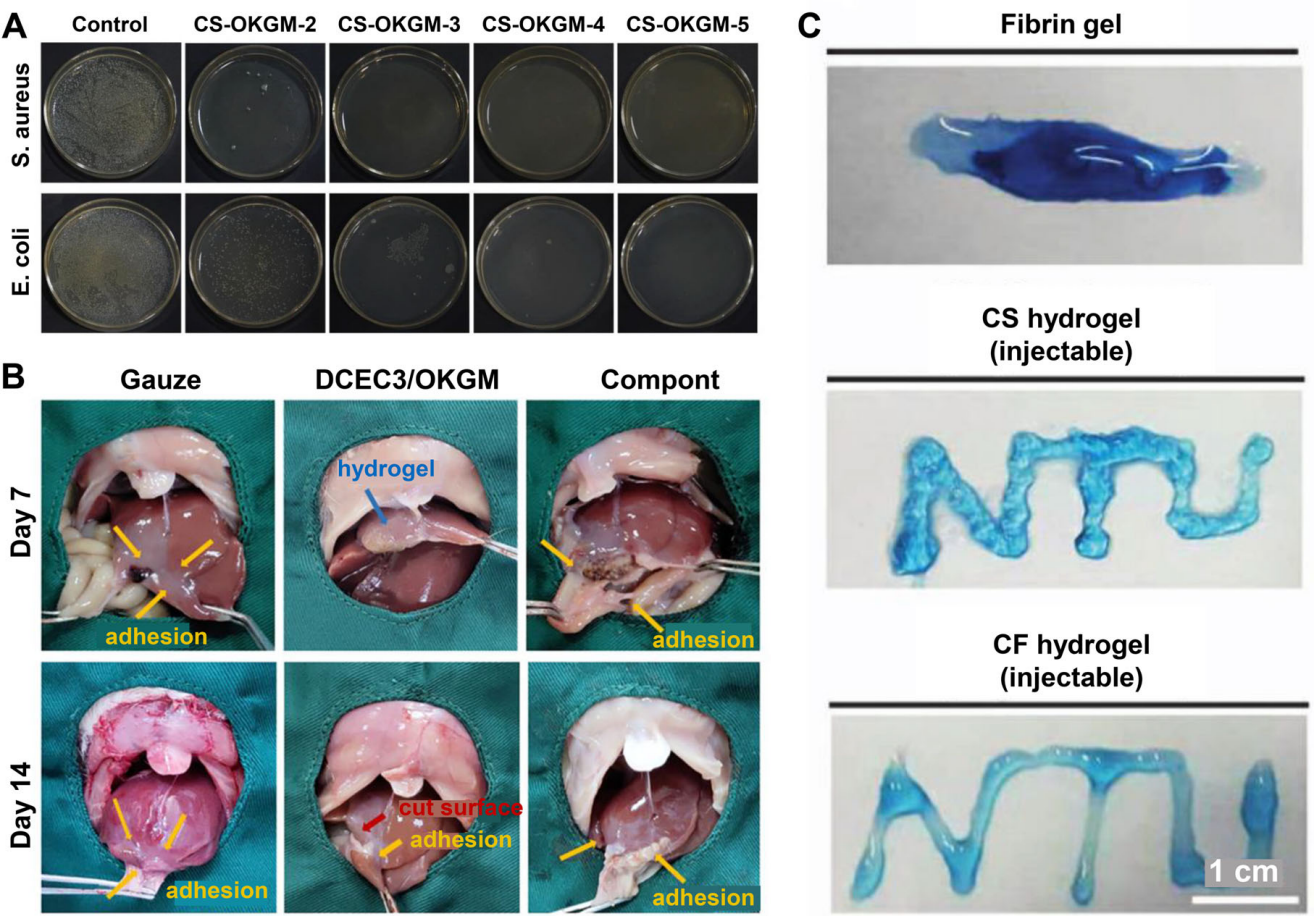
Hydrogels with antibacterial properties utilized for protecting wound injuries. (A) Image of a colony of surviving bacteria on agar plates [169], Copyright©2018, Elsevier. (B) Evaluation of antiadhesion properties of DCEC3/OKGM hydrogels, illustrating their effectiveness in preventing tissue adhesion post‐surgery [170], Copyright©2022, Elsevier. (C) Three hydrogel forms can be seen by Trypan blue staining [87], Copyright©2017, Springer Nature.

Certain clinical treatments frequently result in infections owing to bacterial contamination, necessitating the use of antibiotics for therapeutic intervention [166]. However, antibiotic resistance remains a major challenge. Antibacterial hydrogels are a potential solution for antimicrobial resistance because of their excellent biocompatibility. Their development has resulted in extensive applications in wound healing and other domains [167, 168]. Chen et al. [169] combined CS and OKGM to produce hydrogels with antibacterial properties. The design principle of this hydrogel was based on the reactions of Schiff bases in dynamic covalent bonds. To evaluate their antibacterial efficacies, CS‐OKGM‐2, ‐3, ‐4, and CS‐OKGM‐5 were compared. The findings demonstrated that all the samples exhibited considerable antimicrobial activity against both *Staphylococcus aureus* and *Escherichia coli*. Notably, hydrogels CS‐OKGM‐2, ‐3, ‐4, and CS‐OKGM‐5 demonstrated the ability to eradicate nearly all *S. aureus* cells, while CS‐OKGM‐4 and CS‐OKGM‐5 were responsible for cellular death rates >98% in *E. coli*. In contrast, the CS‐OKGM‐3 and CS‐OKGM‐2 hydrogels achieved 85% and 65% *E. coli* cell mortalities, respectively (Figure 4A).

Based on these results, CS‐OKGM‐4 and CS‐OKGM‐5 emerged as optimal choices for protecting damaged tissues. Wei et al. [170] developed a hydrogel using dodecyl‐modified N‐carboxyethyl CS (DCEC) and OKGM to prevent tissue adhesion following hemostasis. In this study, they evaluated the effectiveness of DCEC3/OKGM hydrogels using gauze and commercial medical gels as controls. Obvious adhesion was demonstrated in rats treated with gauze and commercial adhesives. In contrast, the adhesion of rats treated with DCEC3/OKGM was reduced considerably, indicating good hydrogel performance (Figure 4B). To achieve minimal wound formation while ensuring satisfactory results, Hsieh et al. [87] developed a hydrogel composed of CS and CF that could be injected through a 26‐gauge needle without clotting (Figure 4C).

#### 4.1.2 Bone‐Related Repair

Bone is a hard tissue found in humans and vertebrates that provides structural support to the body and protects internal organs [171]. Conversely, cartilage, is a connective tissue characterized by its supportive role and increased toughness. It lacks blood and lymphatic vessels; instead, nutrients are supplied from the perichondrium through blood vessels and diffuse into the intercellular matrix to nourish cartilage cells. However, factors such as injury, inflammation, and bone disease can lead to bone defects and cartilage damage. Traditionally, the treatment of bone defects relies on bone grafting to facilitate repair [172, 173]. Although autologous cancellous bone transplantation is a common method for healing these defects, limitations arise when the bone defect is extensive, often leading to issues such as bone absorption and nonunion. Furthermore, surgical transplantation is associated with long recovery times, high‐technical demands, and an increased risk of complications, such as fractures and nonunion, which can restrict its biological applications [174]. For cartilage injuries, minor damage may heal with sufficient rest; however, more complex injuries, such as subchondral fractures, typically require arthroscopy and surgical intervention, considerably increasing the risk of infection. Given these challenges, there is an urgent need for innovative methods to treat bone defects and meet clinical needs. Bone tissue engineering technology has become an excellent solution to this problem [175, 176].

**FIGURE 5:**
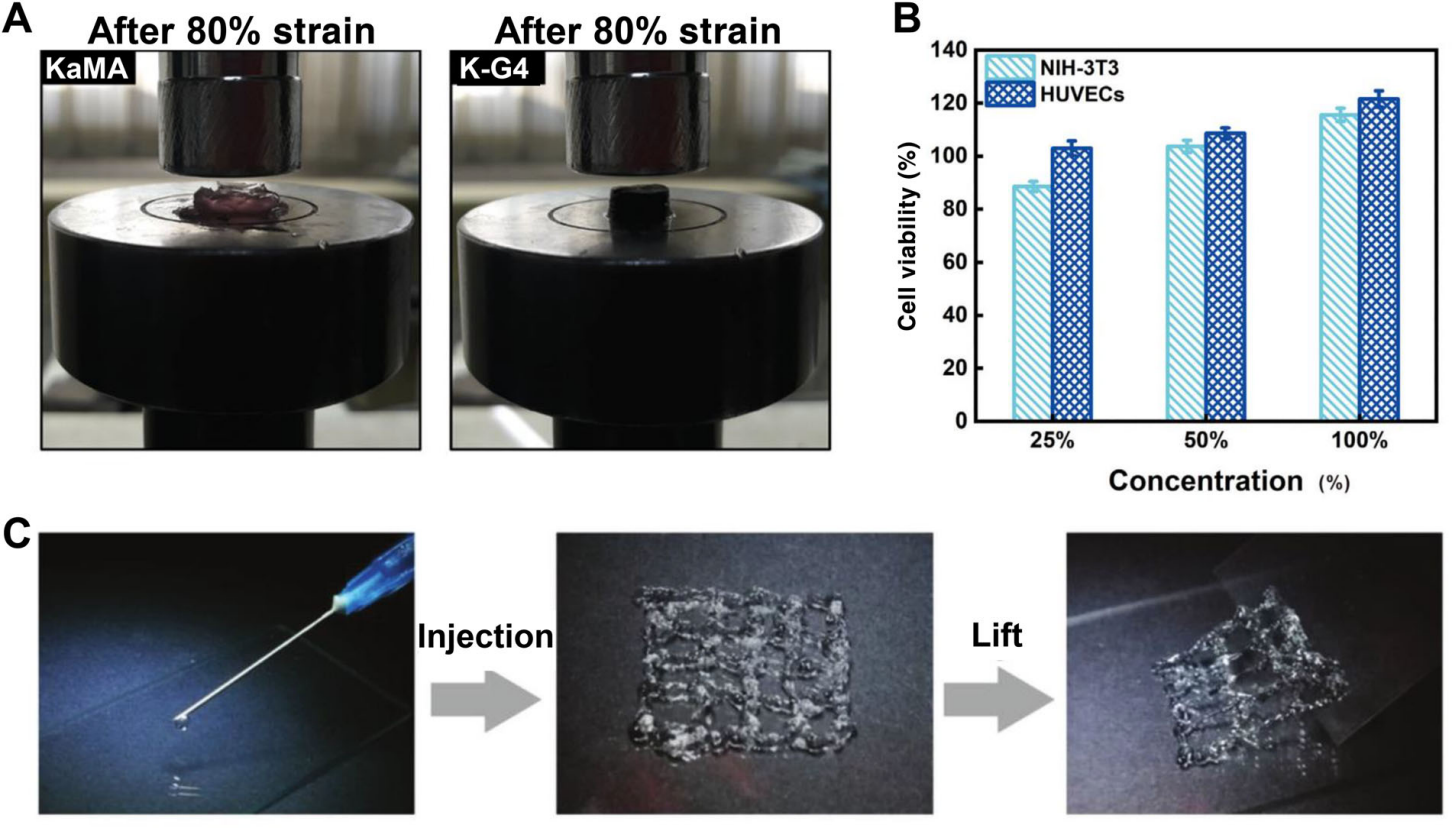
New hydrogels for bone‐related repair. (A) Qualitative images demonstrating the elastomeric behavior of the hydrogels under compressed pressure, highlighting their structural integrity under strain [77], Copyright©2019, Elsevier. (B) The cytotoxicity of hydrogel was determined by Cell Counting Kit‐8 method [177], Copyright©2020, Elsevier. (C) The injection process of hydrogel and the morphology of hydrogel after injection [178], Copyright©2018, John Wiley and Sons.

Mokhtari et al. [77] reported a new hydrogel featuring stable mechanical properties owing to KaMA–dopamine‐functionalized GO. The hydrogel demonstrated remarkable ability to maintain its structural integrity under strain conditions of up to 80%. However, beyond this threshold, the KaMA specimens collapsed and could not revert to their original states. This suggests that it may serve as a support material for bone‐related repair (Figure 5A). Li et al. [177] created hydrogels utilizing N, O‐carboxymethyl CS and oxidized chondroitin sulfate, which exhibited excellent biocompatibility via the Schiff base reaction. The cell viability test results obtained using different hydrogel concentrations showed that the hydrogels were less cytotoxic (Figure 5B). Chen et al. [178] designed a multifunctional hydrogel using BAPEG and DCS. This hydrogel exhibited excellent injectability, as confirmed by the dissociation of its linkages under pressure, which allowed the hydrogel to reshape and flow like a liquid when compressed in a syringe. Upon exiting the syringe, the linkages reformed into solid hydrogels (Figure 5C).

#### 4.1.3 Nerve Repair

**FIGURE 6:**
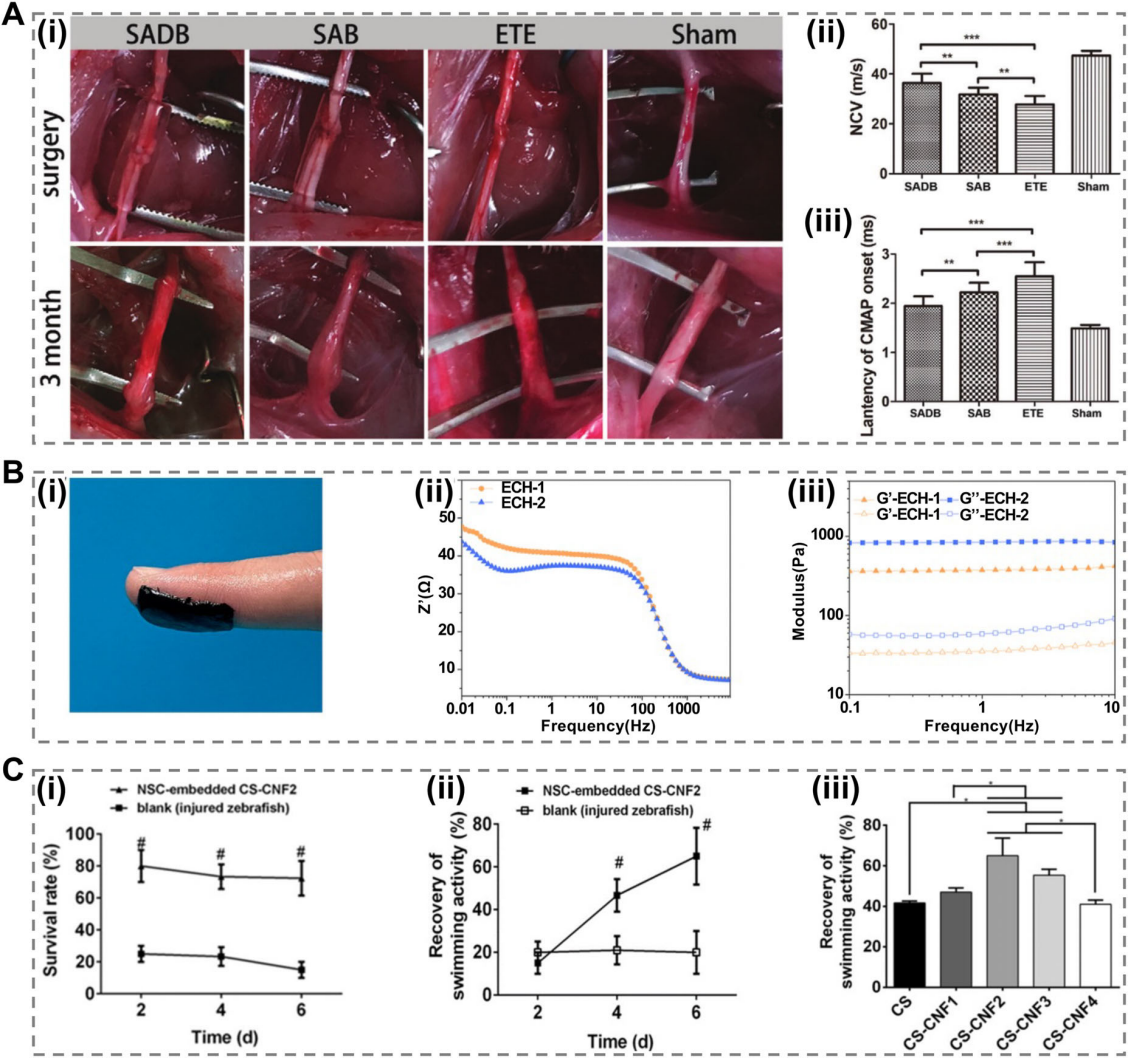
Hydrogels for nerve regeneration and recovery. (A) (i) Observation images of the operative and regenerated nerves. (ii) NCV values of regenerated nerves indicate functional recovery. (iii) The latency period of the onset of regenerative nerve CMAP [180], Copyright©2020, John Wiley and Sons. (B) (i) A photograph demonstrating the adhesive properties of the ECH, with the dressing film applied to a finger. (ii) Bode plots for ECH hydrogels, highlighting their excellent electrical performance. (iii) Evaluation of the mechanistic properties of the ECHs, confirming their suitability for application in nerve repair [181], Copyright©2021, Elsevier. (C) (i) Survival rates of zebrafish treated with NSC‐embedded CS‐CNF2 hydrogels over a period of 6 days. (ii) Swimming activity differences observed in zebrafish receiving the NSC‐embedded CS‐CNF2 hydrogel compared with controls. (iii) Comparison of swimming function recovery across zebrafish treated with various hydrogels containing NSCs, illustrating the effectiveness of different formulations [182], Copyright©2019, Springer Nature.

Owing to the nonregenerative nature of central nerve cells (NSCs), clinical practice primarily relies on supportive and symptomatic treatments. Hydrogels have emerged as promising substitutes for autologous nerve grafts because of their excellent toughness, which makes them suitable for treating nerve injuries [179]. Zhang et al. [180] developed a self‐adhesive bandage designed to wrap around injured nerves, thereby enhancing peripheral nerve regeneration and recovery. This bandage, fabricated using 3D printing technology, incorporates azide‐modified gelatin methacrylate and dibenzyl cyclooctyne‐modified gelatin methacrylate, resulting in a biodegradable self‐adhesive material. Additionally, the flexibility afforded by 3D printing facilitates the loading and directional release of therapeutic drugs for nerve repair. Evaluation results from various groups, including the self‐adhesive drug‐loaded bandages (SADB) group, self‐adhesive bandage (SAB) group, end‐to‐end neurorrhaphy, and sham‐operated (sham) groups, indicated that nerve injuries healed in all groups (Figure 6A(i)). Measurements of the mean nerve conduction velocity (NCV) (Figure 6A(ii)) and latencies of compound motor action potential (CMAP) onset (Figure 6A(iii)) further demonstrated these outcomes. Faster NCVs and shorter CMAP latencies signify better recovery, highlighting the effectiveness of SADB and SAB treatments.

Liu et al. [181] developed a conductive scaffold to treat peripheral nerve injuries by leveraging the benefits of conductivity to enhance nerve repair. Biocompatible electroconductive hydrogel (ECH) dressings consisting of TA and PPY exhibit favorable mechanical properties and electroactivity. These hydrogels offer a soft, self‐healing, and adhesive ECM (Figure 6B(i)). The analysis showed that the charge transfer resistance decreased as the diameter of the semicircle in the electrochemical impedance spectroscopy plots decreased, indicating improved conductivity. Notably, ECH‐2 exhibited superior charge transfer characteristics compared with ECH‐1, as illustrated in the Bode plot (Figure 6B(ii)). Moreover, the storage modulus (elastic modulus *G* *′*) was found to be greater than the loss modulus (viscoelastic modulus *G″*), demonstrating excellent stability and viscoelasticity of the hydrogel (Figure 6B(iii)).

In addition, Cheng et al. [182] investigated the incorporation of low concentrations of CNFs (0.06–0.15 wt%) into pristine CS hydrogels to enhance their self‐healing properties. NSCs were embedded within the hydrogels to aid nerve recovery. The results showed that the CS‐CNF2 hydrogel embedded in the cells was beneficial for the survival of injured zebrafish (Figure 6C(i)). Additionally, comparisons of swimming activities demonstrated differences between zebrafish treated with cell‐embedded CS‐CNF2 hydrogels and those that were not (Figure 6C(ii)), along with variations in outcomes based on different hydrogel compositions over the same duration (Figure 6C(iii)).

#### 4.1.4 Cardiac Repair

Cardiac repair is usually performed using various methods, including medication, surgery, and interventional therapy [183]. In recent years, interventional therapy has opened up a new therapeutic approach for cardiac repair and remarkable progress has been achieved in the field of interventional therapy [184, 185]. Hydrogels are extensively used in cardiac tissue engineering owing to their unique biocompatibility characteristics [186]. Moreover, hydrogels have been tested in clinical trials, effectively demonstrating that they can improve heart function in patients with heart disease [187].

**FIGURE 7:**
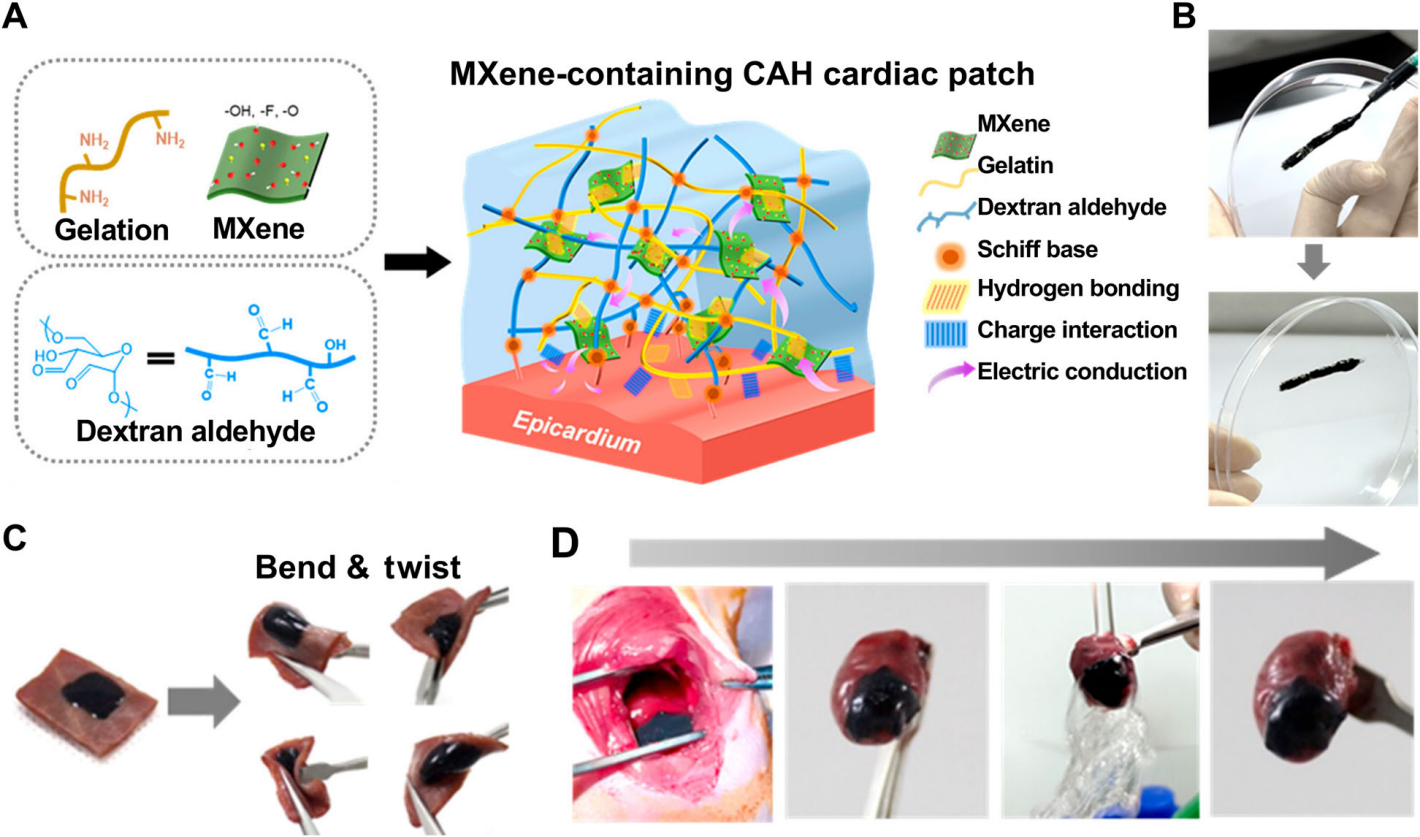
Hydrogels for cardiac tissue engineering. (A) Diagram of hydrogel heart patch. (B) hydrogel photos during and after painting. (C) The hydrogel attached to pig epicardium and epicardium. (D) The hydrogel attached to a living rat heart [188], Copyright©2023 American Chemical Society.

Lee et al. [188] developed an electrically conductive hydrogel heart patch that could stably attach to the heart and play a supporting and electrical activity role, thereby promoting cardiac functional recovery and heart repair. This hydrogel is mainly obtained by the reaction of gelatin and dextran aldehydes through a Schiff base; it subsequently forms tissue adhesion through interactions such as hydrogen bonds with the heart tissue, thus playing a role in protecting the heart (Figure 7A). The hydrogel was coated on a Petri dish; the hydrogel was still stable on the dish after the Petri dish was rotated by 90°, justifying the hydrogel's good adherence properties (Figure 7B). At the same time, the hydrogel was applied onto the epicardial membrane of a pig and rat heart; and regardless of deformation and stretching, the hydrogel maintained its excellent stability and adhesion characteristics (Figure 7C,D).

These hydrogels can be engineered to mimic the mechanical and biological properties of cardiac tissues, making them suitable for application in heart patches and scaffolds for tissue engineering. Wang et al. [189] demonstrated the use of an ECM self‐healing hydrogel for heart repair following myocardial infarction. These results highlighted the potential of self‐healing hydrogels for cardiac repair and tissue engineering.

### 4.2 Drug Delivery

The utilization of self‐healing hydrogels for drug delivery has demonstrated substantial potential, particularly in surmounting formidable barriers, such as the blood–brain barrier (BBB) [190]. The unique structural attributes of self‐healing hydrogels enable them to encapsulate pharmaceutical agents and modulate their release, thereby enhancing drug bioavailability in the central nervous system [191, 192]. For instance, recent studies have indicated that self‐healing hydrogels facilitate more efficient drug transport across the BBB, resulting in more precise drug release and superior therapeutic outcomes [193]. Additionally, self‐healing hydrogels possess substantial promise for ocular administration as they can effectively deliver medications directly to ocular tissues [194]. Their ability to retain moisture and prolong drug release makes them highly suitable for the treatment of a variety of ocular disorders [195]. In preclinical trials, self‐healing hydrogels have been shown to maintain drug concentrations at target sites for extended periods, thereby augmenting the therapeutic efficacy of ocular drug delivery. Self‐healing hydrogels are highly advantageous in the field of 3D bioprinting [196]. Complex biological scaffolds can be fabricated by manipulating the mechanical and bioactive properties of hydrogels. Beyond these applications, self‐healing hydrogels have the potential to enhance the therapeutic efficacy in both drug delivery and tissue engineering [197]. For example, recent advancements in 3D bioprinting have shown that self‐healing hydrogels can be employed to generate functional tissues and organs precisely.

### 4.3 Biosensing

Hydrogels have recently emerged as indispensable materials in biosensing. Owing to their exceptional biocompatibility and unique mechanical properties, diverse arrays of responsive hydrogels can be engineered through specific physical and chemical modifications [198, 199]. These hydrogels maintain the activity of biomolecules, enabling the detection and monitoring of specific biomarkers through their interactions with enzymes, antibodies, and other biomolecules [200]. Liu et al. [201] developed a natural hydrogel sensor by utilizing ionic compounds, such as LiCl and β‐sodium glycerophosphate, in conjunction with the high viscoelasticity and superior biocompatibility of gelatin. This hydrogel sensor could detect human movements and respiration.

As a natural hydrogel, CS has attracted considerable attention for the preparation of biocomposites owing to its excellent mechanical properties. Castellanos et al. [202] developed a plasma hydrogel using CS, obtained through noncovalent interactions between CS and diethyl latinate. This CS hydrogel exhibited high biocompatibility; it provides research ideas for creating new biomaterials with responsive biological signals. Ren et al. [203] designed a nanofiber hydrogel response sensor. The advantage of this sensor is that the high‐mechanical‐strength hydrogel obtained by crosslinking TPU‐PVAc@AgNP/MXene nanofibers with formic acid has an ECM structure that provides the sensor with high sensitivity and is suitable for the detection of various fine movements, particularly underwater movements. In summary, new hydrogel sensors with higher sensitivity can be developed by exploiting the mechanical properties of hydrogels. The sensing and response abilities of hydrogels provide them with application advantages in the fields of biomedicine, medicine, and environmental monitoring.

## 5 Challenges and Perspectives

In recent years, extensive progress has been achieved in the preclinical and clinical evaluations of self‐healing hydrogels. These studies have explored the potential of self‐healing hydrogels for various biomedical applications, including tissue repair, drug delivery, and biosensing. The following section summarizes the relevant preclinical animal experiments and clinical trials and highlights their mechanisms of action and outcomes (Table 3).

**TABLE 3:** Summary of preclinical and clinical studies on self‐healing hydrogels.

Application area	Mechanism of action	Key findings	NCT number, phase [204]	References
Wound healing	Dynamic cross‐linking (catechol‐borate bonds)	Superior wound healing compared with commercial dressings	NCT05378997 (Ph I), NCT04601532 (Ph IV), NCT05411484 (Ph III), NCT03564795 (NA)	[163], 205]
	Cysteine‐specific dissolution	Rapid drug release and effective wound healing		[133, 206, 207]
Bone‐related repair	Mechanical robustness (κ‐carrageenan‐GOPD)	Structural integrity under strain, suitable for bone repair	NCT05398341 (not given), NCT04379700 (NA), NCT05051332 (Ph III)	[77, 208]
	Biocompatibility (N, O‐CMC/OCS)	Excellent biocompatibility and cytocompatibility		[177, 209]
Nerve repair	Self‐adhesive properties (N3‐GelMA/DBCO‐GelMA)	Significant nerve function recovery	NCT03940963 (NA), NCT04865679 (NA)	[180, 210]
	Conductivity (TA/PPY)	Enhanced nerve regeneration		[181, 211]
Cardiac repair	Electrical conductivity and adhesion	Promoted cardiac function recovery	NCT04396899 (Ph I, Ph II)	[188, 212]
	Extracellular matrix self‐healing	Improved cardiac function and tissue regeneration		[189, 213]
	Clinical trial (cardiac ECM hydrogel)	Safety and efficacy in myocardial infarction patients		[187]

The advent of self‐healing hydrogels has led to considerable advancements in tissue repair and regeneration, offering a dynamic and responsive approach for wound management, bone regeneration, nerve regeneration, and cardiac repair [214, 215, 216, 217]. These materials emulate the innate healing mechanisms of the body, providing sustained drug release and the ability to recover autonomously from mechanical damage [218, 219, 220]. Despite their promising potential, self‐healing hydrogels face several challenges that must be addressed to facilitate their transition from laboratory research to clinical applications.

The tradeoff between mechanical strength and self‐healing efficacy remains a major challenge [221, 222]. Hydrogels based on dynamic covalent bonds, such as disulfide and Schiff base bonds, often exhibit robust self‐healing capabilities, but may lack the mechanical strength required for high‐stress applications. Fu et al. [163, 219] developed a supramolecular hydrogel that leveraged ion–dipole interactions to demonstrate self‐healing properties. However, despite its effective self‐healing capacity, the mechanical strength of this hydrogel is insufficient for applications requiring high‐tensile strengths [223]. To reconcile these two critical attributes, future research must focus on optimizing the chemical structure of hydrogels. The incorporation of reinforcing agents or novel crosslinking strategies may be necessary to enhance the mechanical performance without compromising their self‐healing capabilities [224].

Hydrogels used in clinical settings must exhibit excellent biocompatibility and biodegradability [225]. Natural polysaccharides, such as CS and HA, are frequently utilized because of their superior biocompatibility; however, their mechanical properties often require enhancement [226, 227]. For example, HA‐based hydrogels, which closely resemble the ECM, are commonly used for tissue engineering [228]. Nevertheless, their inherently low‐mechanical strength requires chemical modification to improve durability [229]. In addition, the degradation rate of hydrogels must be meticulously controlled to match the tissue regeneration process, thereby preventing the material from remaining in place longer than necessary, potentially causing complications [230]. This necessitates the development of customized degradation profiles and a thorough understanding of the biological context in which the hydrogel is applied.

This stringent regulatory landscape poses considerable challenges for the clinical application of self‐healing hydrogels. The rigorous United States Food and Drug Administration requirements concerning the safety, efficacy, and biocompatibility of novel biomaterials present multiple hurdles [231, 232]. For example, despite advances in clinical trials, a cell extract‐based hydrogel intended for cardiac repair has faced a protracted approval process, highlighting the complexity of regulatory compliance [187]. Moreover, the diverse array of materials generated through multifaceted functional modifications of hydrogels complicates their approval and classification. This situation underscores the need for a comprehensive and well‐defined regulatory framework that ensures material safety and efficacy, while facilitating timely integration into clinical practice.

Numerous intriguing research directions have emerged to overcome these challenges and unlock the full potential of self‐healing hydrogels. Biohybrid materials that integrate hydrogels with biological molecules offer enhanced sensitivity to pathological conditions and superior biocompatibility [233]. For example, a combination of hydrogels with ECM components can yield materials with improved performance in tissue engineering and drug delivery [234]. Angiogenesis, a critical phase in tissue regeneration, can be effectively promoted by copper ion (Cu^2^⁺)‐doped HA hydrogels [235]. However, it is essential to carefully control high Cu^2^⁺ concentrations to avoid cytotoxicity and adverse effects on cell viability. The development of these biohybrid materials has advanced the development of hydrogels capable of actively facilitating healing.

The production of hydrogels is undergoing a transformative revolution, largely owing to advancements in manufacturing processes, such as 3D printing [196]. This technology enables precise fabrication of complex structures that meet specific medical requirements. For instance, successful 3D printing of CS‐ and HA‐based hydrogels into scaffolds for tissue engineering demonstrates how this technique can be utilized to create customized implants [236, 237]. In addition, the development of conductive self‐healing hydrogels, such as those based on PPY, has opened new avenues for applications in bioelectronics and nerve repair [238]. These advancements underscore the critical importance of integrating hydrogel research with modern manufacturing techniques to produce materials that are practical and perfectly tailored to their intended applications.

Self‐healing hydrogels are currently underutilized because of their prohibitively high‐production costs [239]. To scale‐up production and reduce costs, it is imperative to utilize low‐cost raw materials and optimize manufacturing processes. Hydrogels derived from natural polysaccharides are promising alternatives because of their cost effectiveness and ease of production [240]. By streamlining the production processes without compromising the quality or efficacy of the hydrogels, costs can be reduced considerably, thereby enhancing their accessibility for clinical applications. This may involve the development of novel synthesis methods or leveraging renewable resources to minimize the overall production expenses.

Although laboratory studies on self‐healing hydrogels have yielded promising results, several challenges must be addressed before these advancements can be translated into clinical applications [241]. Clinical trials of self‐healing hydrogels must be meticulously designed and executed to ensure their safety and efficacy. These trials should consider patient variability, disease type, and specific treatment requirements [204]. For example, in applications involving bone repair, the mechanical properties and bioactivities of hydrogels must be tailored to satisfy the demands of bone tissue regeneration [242]. Additionally, clinical trials require long‐term monitoring and efficacy evaluations to confirm that the hydrogel continues to perform as intended. This requires a comprehensive approach to clinical testing that incorporates the assessment of patient outcomes over both short‐ and long‐term periods.

Another major challenge lies in scaling up the production of self‐healing hydrogels from the laboratory to industrial settings. The widespread clinical application of these materials is currently limited by difficulties associated with the upscaling of many existing manufacturing processes. For example, hydrogels based on dynamic covalent bonds often require intricate production procedures that are difficult to replicate on a large scale [243]. To commercialize these innovative materials, more efficient and scalable manufacturing methods must be developed [244]. This may necessitate the development of new technologies or the adoption of continuous manufacturing techniques to achieve large‐scale production while maintaining the consistency and quality of hydrogels.

When discussing the future of self‐healing hydrogels, it is imperative to acknowledge the multifaceted challenges that they face. Despite their considerable potential in biomedicine, issues related to their biocompatibility, biodegradability, production costs, and regulatory barriers must be addressed. For instance, the optimization of the biocompatibility and biodegradability of hydrogels is essential to ensure their safe and effective use in the human body [245]. However, to render these materials commercially viable and accessible for clinical applications, production costs must be reduced and scalability must be enhanced. To overcome these obstacles and enhance the hydrogel performance, future research should focus on developing innovative approaches that will ultimately pave the way for broader healthcare utilization. The field of self‐healing hydrogels is expected to advance considerably if these challenges are confronted directly, and novel enhanced solutions are provided for a variety of biomedical applications.

## 6 Conclusions

As an innovative biomaterial, self‐healing hydrogels are gradually breaking through the limitations of traditional materials. In recent years, the research team has made remarkable progress in material design, performance optimization and application scenarios through multidisciplinary strategies, but many challenges remain. The synergistic improvement of mechanical properties and self‐healing ability makes hydrogels more similar to the mechanical properties of human tissues. Second, self‐healing and non‐self‐healing hydrogels were combined into a composite gel system through three‐dimensional crosslinking grids, which endowed the material with dynamic repair ability and excellent biocompatibility, providing a new idea for the design of functional implant materials. In addition, expanding the application of hydrogels in the field of nano will further realize the multifunction of hydrogels. At the same time, self‐healing hydrogel manufacturing technology is constantly being innovated, such as the introduction of 3D printing technology has laid the foundation for the personalized customization of medical hydrogels, and solved the preparation problem of complex structure hydrogels.

Although the future research prospects of self‐healing hydrogels are considerable, there are still bottlenecks in clinical transformation. Most of the existing studies remain in the laboratory stage, and the obstacles facing clinical application include the long‐term biosafety of hydrogel materials, and the metabolic pathway of hydrogel materials in human body is still unclear. Second, the environmental adaptability of self‐healing hydrogels still needs to be further explored, for example, the humidity and PH of the wound environment may affect the performance of hydrogel materials. In addition, the intelligence of self‐healing hydrogels still needs to be explored, and the combination of hydrogels and AI technologies may provide new ideas for the precision treatment of hydrogels. In the future, with the introduction of materials genomics, artificial intelligence‐assisted design and other technologies, hydrogels are expected to achieve the leap from “bionics” to “superbiology,” becoming the core material in tissue engineering, flexible electronics and other fields, and ultimately promoting the transformation of medical mode from “passive treatment” to “active repair”.

Self‐healing hydrogels represent an innovative class of biomaterials that hold immense promise for tissue regeneration and repair. Their ability to deliver drugs continuously, respond to environmental stimuli, and mimic biological healing processes has opened new frontiers in biomedical engineering. However, achieving an optimal balance among mechanical strength, biocompatibility, biodegradability, and self‐healing efficiency is a formidable challenge. Although extensive advancements have been made in the development of self‐healing hydrogels, much remains to be achieved. Additional research studies are required to enhance the physiological performance of these materials, refine their properties, and ensure their safety and efficacy in clinical settings. As our understanding of these materials deepens, their potential applications continue to expand, potentially revolutionizing medical approaches for tissue regeneration and repair. With continued study and development, self‐healing hydrogels have the potential to become the cornerstone of biomaterials, offering creative solutions to some of the most intractable problems in the medical field.

## Author Contributions

Zhongxia Wang, Haozhen Ren, Decai Yu, and Xinhua Zhu conceptualized the idea and structured the article. Lingling Xue, Ran An, Junqi Zhao, and Mengdi Qiu conducted literature reviews and composed the manuscript. Lingling Xue and Junqi Zhao contributed to the writing process. Ran An, Zhongxia Wang, Decai Yu, and Xinhua Zhu contributed to the revised writing. All authors have read and approved the final manuscript

## Ethics Statement

The authors have nothing to report.

## Conflicts of Interest

The authors declare no conflicts of interest. The authors declare that they have no conflict of interest.

## Data Availability

The authors have nothing to report.
